# Surface Topography of PLA Implants Defines the Outcome of Foreign Body Reaction: An In Vivo Study

**DOI:** 10.3390/polym15204119

**Published:** 2023-10-17

**Authors:** Elena Ivanova, Alexey Fayzullin, Nikita Minaev, Irina Dolganova, Natalia Serejnikova, Elvira Gafarova, Mark Tokarev, Ekaterina Minaeva, Polina Aleksandrova, Igor Reshetov, Peter Timashev, Anatoly Shekhter

**Affiliations:** 1 Institute for Regenerative Medicine, Sechenov First Moscow State Medical University (Sechenov University), 8-2 Trubetskaya St., Moscow 119991, Russia; ivanova_e_i_1@staff.sechenov.ru (E.I.); fayzullin_a_l@staff.sechenov.ru (A.F.); serezhnikova_n_b@staff.sechenov.ru (N.S.); gafarova_e_r@staff.sechenov.ru (E.G.); tokarev_m_v@staff.sechenov.ru (M.T.); timashev_p_s@staff.sechenov.ru (P.T.); 2 B.V. Petrovsky Russian Research Center of Surgery, 2 Abrikosovskiy Lane, Moscow 119991, Russia; 3 Institute of Photon Technologies of FSRC “Crystallography and Photonics” RAS, Troitsk, Moscow 108840, Russia; minaevn@gmail.com (N.M.); minaeva.e.d@bk.ru (E.M.); 4 Osipyan Institute of Solid State Physics of the Russian Academy of Sciences, 2 Osipyan St., Chernogolovka 142432, Russia; dolganova@issp.ac.ru; 5 Prokhorov General Physics Institute of the Russian Academy of Sciences, 38 Vavilova St., Moscow 119991, Russia; aleksandrovapolina98@gmail.com; 6 L.L. Levshin Institute of Cluster Oncology, Sechenov First Moscow State Medical University (Sechenov University), 8-2 Trubetskaya St., Moscow 119991, Russia; reshetov_i_v@staff.sechenov.ru; 7 World-Class Research Center “Digital Biodesign and Personalized Healthcare”, Sechenov First Moscow State Medical University (Sechenov University), 8-2 Trubetskaya St., Moscow 119991, Russia

**Keywords:** microtextured implants, implant, polylactide, foreign body reaction, fibrosis, peri-implant fibrosis, peri-implant capsule, collagen

## Abstract

The formation of a dense fibrous capsule around the foreign body and its contracture is the most common complication of biomaterial implantation. The aim of our research is to find out how the surface of the implant influences the inflammatory and fibrotic reactions in the surrounding tissues. We made three types of implants with a remote surface topography formed of polylactide granules with different diameters: large (100–200 µm), medium (56–100 µm) and small (1–56 µm). We placed these implants in skin pockets in the ears of six chinchilla rabbits. We explanted the implants on the 7th, 14th, 30th and 60th days and performed optical coherence tomography, and histological, immunohistochemical and morphometric studies. We examined 72 samples and compared the composition of immune cell infiltration, vascularization, the thickness of the peri-implant tissues, the severity of fibrotic processes and α-SMA expression in myofibroblasts. We analyzed the scattering coefficient of tissue layers on OCT scans. We found that implants made from large granules induced a milder inflammatory process and slower formation of a connective tissue capsule around the foreign body. Our results prove the importance of assessing the surface texture in order to avoid the formation of capsular contracture after implantation.

## 1. Introduction

Regenerative medicine is a rapidly developing field of medicine that involves the restoration of lost functions or structures of tissues and organs of the human body. One of the fundamental directions of regenerative medicine is the creation of scaffolds and tissue engineering structures for regeneration. The most important limiting factor for the widespread translation of the results into clinical practice is the immune response to an implant recognized as a foreign body. The problem of immune response to implants is currently addressed in two ways: using the patient’s own (autologous) cells and implanting cell-free biomaterial constructs [1,2,3,4,5,6]. A typical response of the recipient tissue to an implant is peri-implant fibrosis, the formation of a connective tissue capsule around the foreign material isolating it from the surrounding tissues. However, significant fibrosis of the connective tissue often occurs around the implant. The main sign of fibrosis is an increase in the number or the synthetic activity of fibroblasts (hyperplasia and hypertrophy), which leads to excessive accumulation and contraction of collagen and other components of the extracellular matrix. Different cellular components are involved in this process at different stages of the foreign body reaction. Leukocytes, macrophages and fibroblasts enact a biological response to the implant during the first few weeks after the operation. Later, multinucleated giant cells of foreign bodies and myofibroblasts form granuloma and connective tissue capsules [7]. These two types of cells control the long-term immune response to the implant and define the choice of biological program between resorption and isolation of the implant [8,9,10,11,12].

Chemical or physical properties of the implant influence inflammatory and fibrotic responses. Changes in the chemical composition of the implant or its surface and simultaneous modulation of physical parameters, such as surface hydrophilicity, porosity, stiffness, anisotropic roughness and topography, have an effect on angiogenesis, inflammatory and fibrotic reactions around the implant [13,14]. Some authors suggest that the physical characteristics of the implant affect myofibroblasts, which are involved in the formation of connective tissue and its fibrosis. The formation of fibrous connective tissue may lead to implant degradation and surgical revision [13]. Thick materials (more than 100 µm) cause more severe fibrosis and a more severe inflammatory response to the foreign body. Ward et al. investigated the effect of material size by comparing the recipient response to the implantation of cylindrical polyurethane implants 300 µm and 2000 µm in size. They found that an increase in the thickness of the implanted material resulted in stronger foreign body reactions and the formation of a thicker layer of fibrous connective tissue around the implant [15]. The microtextural profile of the surface of single-material implants is also an important factor in the immune response and fibrotic reactions [13,14,15,16,17]. It was shown that two weeks after the implantation of spheres made of alginate hydrogel with different diameters ranging from 0.3 mm to 1.9 mm into the abdominal cavity of mice, the spheres with a smaller diameter led to more severe fibrotic reactions [17]. Therefore, altering the characteristics of the implant surface is one of the most important factors that can improve tissue integration and reduce the incidence and severity of implant damage by inflammatory cells and the development of fibrosis [13,14,15,16,17].

In this article, we want to evaluate how cellular and especially fibrotic reactions depend on the diameter of the granules of the material of which the implant was made. We used the rabbit ear skin pocket model for the reproducible formation of peri-implant capsules. This study focuses on implant-tissue biology between synthetic polymers and soft tissues. An additional aim of the study is to investigate whether we can evaluate biodegradation in situ using optical coherence tomography (OCT).

## 2. Materials and Methods

### 2.1. Preparation of Implants

The implant material was polylactide (PLA) PDL04 (Purasorb, Corbion). It was initially produced in the form of 3–5 mm granules. The polymer granules were frozen (−20 °C) and then mechanically ground for 30 s with an interval of 15 min (~30 grinding cycles). Then, the polymer powder was sieved through a set of 200, 100 and 56 µm sieves. The resulting ground polymer was separated into individual fractions. We used three sets of polymer powders to form three types of implants containing polymer granules ranging in size: 100–200 µm (large fraction), 56–100 µm (medium fraction) and < 56 µm (small fraction). A surface-selective laser sintering method was used to form the implants [18,19]. Distilled water was used as a thermal sensitizer to localize the sintering process of polymer particles on their surface. The experimental samples were formed into 10 mm diameter disks; the height for each set of samples was different and depended on the thickness of the powder layers used (average about 1 mm). The powders were laser processed using an LscanH-10 galvo scanning system (Ateko-TM, Moscow, Russia) with an SL-2000-100-160 F-theta objective (Ronar-Smith, Singapore) and a TLM-3 infrared Tm CW fiber laser source (NTO “IRE-Polus”, Fryazino, Moskovskaya oblast, Russia). The laser source was operated at λ = 1.9 µm with a maximum average power of P = 3 W. The laser system generated a laser spot with a diameter of d~280 μm and moved it at a speed of 15 mm/s, which ensured that only the surface areas of the polymer powder were sintered (<2 µm in distance from the surface of polymer particles) without significant thermal transformation of the polymer mass; heating was predominantly on the surface of the microparticles. The laser sintering temperature was controlled with a FLIR A655SC (FLIR Systems, Wilsonville, OR, USA). The temperature load on the polymer mass did not exceed 70 °C. 

### 2.2. Characterization of Implants

**Figure 1:**
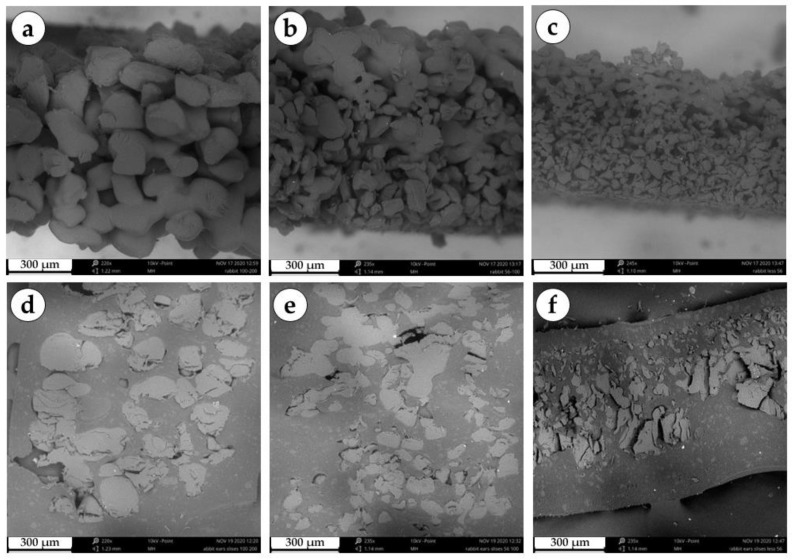
Scanning electron microscopy of a series of granular implants. (**a**) Edge of a three-dimensional large granule disc. (**b**) Edge of a three-dimensional medium granule disc. (**c**) Edge of a three-dimensional small granule disc. (**d**) Section of a three-dimensional large granule disc. (**e**) Section of a three-dimensional medium granule disc. (**f**) Section of a three-dimensional small granule disc.

We studied the surface texture of the sintered implants using a Phenom ProX scanning electron microscope (Phenom World, Eindhoven, the Netherlands) at an accelerating voltage of 10 kV (Figure 1). For the large granule implants, the following structure sintering parameters were used: horizontal fill density 3 lines/mm, laser spot movement speed 15 mm/s and power 100% (~3 W). The disks consisted of three layers with thickness of 300 µm, 300 µm and 400 µm. The sintering parameters for the medium granule implants were 3 lines/mm (horizontal fill density), 18 mm/s (laser spot movement speed) and 100% (power, ~3 W). The disks consisted of three layers with thickness of 400 µm, 400 µm and 500 µm. Horizontal fill density 3 lines/mm, laser spot movement speed 20 mm/s and power 100% (~3 W) were used to produce small granule implants. The disks consisted of four layers with thickness of 200 µm, 200 µm, 200 µm and 300 µm.

**Figure 2:**
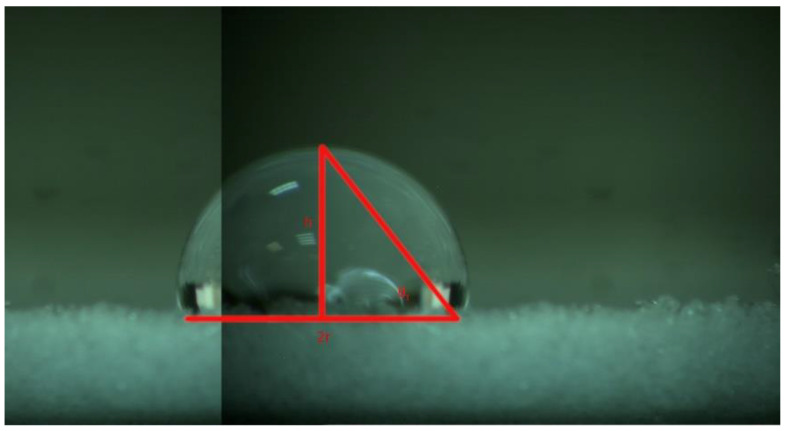
Photograph of a drop of distilled water placed on the surface of a three-dimensional small granule disc. h is the height, and r is half the length of the line of contact between the drop and the material. θ_1_ = tan − 1(h/r).

We calculated the contact angle by the semi-angle method for the used polymer (Figure 2). The contact angle was expressed as θ = 2θ_1_.

**Figure 3:**
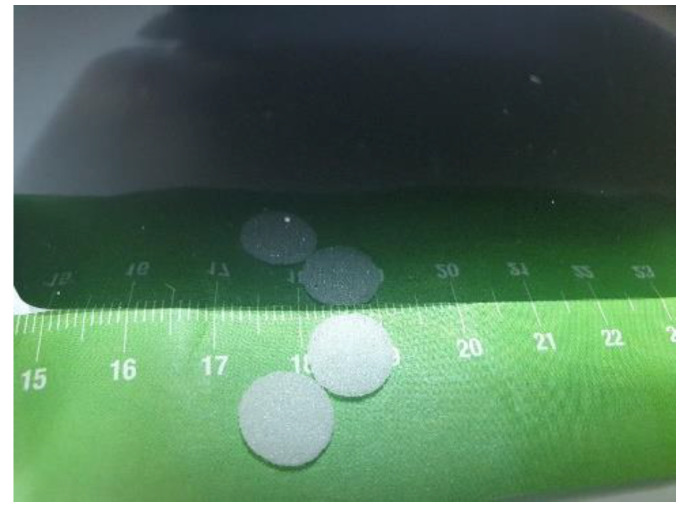
Macrophotograph of a large granule disc.

There was no significant visual difference between the implants of different granules (Figure 3). However, small granule discs were slightly shinier when put under the light.

### 2.3. Surgical Procedures

The required number of animals was calculated with the ClinCalc.com sample size calculator. Based on the preliminarily estimated thickness and number of blood vessels in peri-implant tissues, the required sample size was six, with a confidence level of 95% and a 5% margin of error. The experiment on six 6-month-old chinchilla rabbits (males, 2–2.5 kg, of the same litter) was approved by the Local Ethics Committee of Sechenov University (protocol No. 06-19/15.05.2019). The rabbits were kept under standard vivarium conditions, one animal per cage. The animals were provided with complex granulated laboratory chow and constant access to water. We performed two operations on each rabbit. During the first operation, six PLA discs (two of each type) were implanted in skin pockets of one ear. The second operation was held on day 7 for three rabbits and day 30 for the other three rabbits. The first three rabbits were euthanized on day 14. The latter three rabbits were euthanized on day 30. Before the operations, the animals were anesthetized by intramuscular injection of ZOLETIL 100 solution (VIRBAC, Carros, France; 6 mg/kg of animal body weight), supplemented by local anesthesia of the surgical field with 0.5% novocaine solution.

Granular polylactide implants of three different sizes were fixed subcutaneously with 3-0 Prolene surgical suture (Ethicon, Bridgewater, NJ, USA) in 1.5 × 1.5 cm skin pockets on the ventral side of rabbit ears (between the dermis of the skin and the perichondrium of the ear cartilage). Two small granule implants, two medium granule implants and two large granule implants were placed in skin pockets of each ear. The implantation sites were selected according to the hypertrophic scar model [20]. We followed the protocol for modeling peri-implant fibrosis in rabbit ears [21]. The implants were placed at least 1.5 cm from the marginal auricular artery and from each other. Post-operative antibiotic therapy was performed by intramuscular injections of Baytril 5% (Bayer, Leverkusen, Germany) at a daily dose of 5 mg of enrofloxacin per 1 kg of the animal’s body weight for five days after surgery.

The rabbits were euthanized by injection of a solution of Zoletil 100 (Vibrac, Carros, France; 60 mg/kg animal body weight) after 7, 14, 30 and 60 days. The sites of implantations were dissected with the surrounding tissues approximately 2–3 mm from the edges of the original wound along with the implanted materials. The excised tissues were fixed in 10% neutral buffered formalin for histological analysis.

### 2.4. Histological Analysis

The samples were fixed in formalin for 24 h, then underwent standard histological processing and were embedded into paraffin blocks. Four μm thick sections of the tissue samples were stained with hematoxylin and eosin (H&E), Mallory’s trichrome stain and Picrosirius red (PSR) for the detection of collagen fibers. A LEICA DM4000 B LED microscope, equipped with a LEICA DFC7000 T digital camera running under the LAS V4.8 software (Leica Microsystems, Wetzlar, Germany) was used for the examination and imaging of the samples. The specimens were studied using standard (for H&E, Mallory and PSR stained samples) and polarized light (PSR stained samples) microscopies.

### 2.5. Immunohistochemical Analysis (IHC)

For the immunohistochemical study, four μm thick sections of the formalin-fixed paraffin-embedded tissue samples were deparaffinized, incubated with 3% H_2_O_2_ for 10 min and underwent heat-induced epitope retrieval (pH 6.0 sodium citrate buffer, 30 min in 80 °C water bath); they were additionally blocked with Background Block (Cell Marque, Rocklin, CA, USA), incubated with mouse monoclonal primary antibodies against α-smooth muscle actin (α-SMA) (A2547, Merck, Rahway, NJ, US, diluted 1:400) and detected by HRP-conjugated secondary goat antibodies (G-21040, Invitrogen, Carlsbad, CA, USA, diluted 1:1000) and diaminobenzidine (DAB) with hematoxylin counterstaining.

### 2.6. Morphometry

Morphometric analysis of the histological samples was performed by two blinded pathologists. The discrepancies in their results were resolved by a third pathologist. This pathologist had knowledge of the groups to which the samples’ belonged and wrote the histological report for each study group.

The thickness of the peri-implant tissue was measured in each histological sample at five sites located ~400 µm apart at the center of the implantation site. Measurements were taken from the inner surface of the epidermal–dermal junction to the upper surface of the perichondrium of the cartilage plate. The relative area of the implants was measured in the central parts of each histological slide by segmentation of white pixel areas using ImageJ software (Bethesda, MD, USA). The threshold color function using the Otsu method was used for the creation of binary images of the regions of interest. The binary image total area of white pixels was measured in µm^2^, divided by the cross-sectional area of intact implants of the corresponding length, which is equal to 3.5 × 10^5^ µm^2^ (considering that the original height of the implants was 1 mm, the diameter was 10 mm and the average length of the intact implant fragment, visible at the selected microscope magnification, was 350 µm) and multiplied by 100%. Blood vessel density was evaluated at ×200 magnification in 5 representative fields of view. The results of the blood vessel density analysis were presented as average values per 1 mm^2^.

The intensity of the morphological features and α-SMA expression were evaluated in the entire peri-implantation region. The intensity of the morphological findings was assessed by a semi-quantitative score system, from 0 to 3 (Table A1, Table A2, Table A3, Table A4, Table A5, Table A6, Table A7, Table A8, Table A9 and Table A10 in Appendix A).

### 2.7. Optical Coherence Tomography

OCT scanning was performed using the endoscopic OCT system OCT1300Y developed in the Institute of Applied Physics RAS (Nizhny Novgorod, Nizhny Novgorod Oblast, Russia). The utilized device employed laser radiation with a central wavelength of 1300 nm and an average power of 0.6 mW. It acquired B-scans (2D depth images of 256 × 400 pixels) of the samples with optical penetration depth near 1.0 mm. The resolution in the air was 20 µm and 24 µm in the lateral and axial directions respectively. The visualization area was 2 mm in lateral coordinates and 1–2 mm in the axial direction.

Tissue images were accumulated for three types of implants. The analysis was performed on a set of 18 OCT scans for each type, obtained on the day of explantation and on the 7th, 14th, 30th and 60th day after implantation. A database of 72 scans was formed for each type of implant.

Scan preprocessing included image filtering with a smoothing filter to reduce the effect of speckled noise.

For the obtained initial OCT scans, we performed an analysis of the scattering coefficient of various layers, which we distinguished in each specific case. Subsequently, we obtained average values of the scattering coefficients for each A-scan (for three types of implants made of granules with different sizes and at five time points: 0, 7, 14, 30 and 60 days after implantation).

### 2.8. Statistical Analysis

Statistical analysis of the data was performed with the standard software package GraphPad Prism, version 8.00 for Windows (GraphPad Software, Inc., San Diego, CA, USA). The distribution of the quantitative data was checked by Shapiro–Wilk’s normality test. The intergroup differences were analyzed using the two-way ANOVA followed by Tukey’s multiple comparison test. The differences in the histological scores were evaluated by the Kruskal–Wallis test followed by Dunn’s multiple comparison test. The statistical analysis results were presented as connecting lines with error bars of the mean values and standard deviation (SD) or interleaved bars of median values and interquartile ranges; *p*-values ≤ of 0.05 were considered statistically significant. 

## 3. Results

### 3.1. Histological and Immunohistochemical Analyses

In the samples of all the study groups and time points, the regions of implantation were covered with stratified squamous epithelium. The dermis was moderately infiltrated with lymphocytes. The implant material was observed as round empty holes of different diameters under the dermis. The material particles fused with each other and formed cavities. There was no evidence of dystrophy in the cartilage plate and perichondrium. The thickness, density and architectonics of collagen bundles in the peri-implant capsule and the immune infiltration differed between the groups and changed over time. 

**Figure 4:**
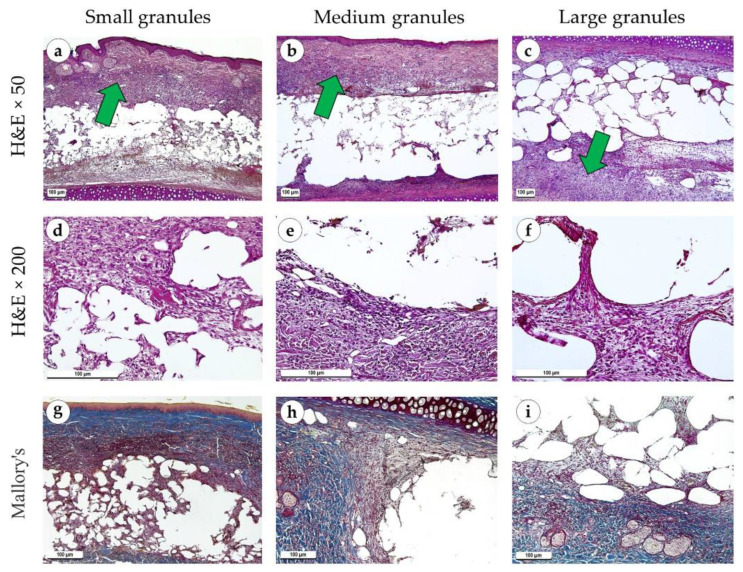
Sites of implantation of discs made of small (**a**,**d**,**g**), medium (**b**,**e**,**h**) and large (**c**,**f**,**i**) granules, 7 days after surgery. (**a**–**c**) H&E stain, magnification ×50. (**d**–**f**) H&E stain, magnification ×200. (**g**–**i**) Mallory’s trichrome stain, magnification ×100. Scale bars are 100 µm. Granulation tissue is marked with green arrows.

**Figure 5:**
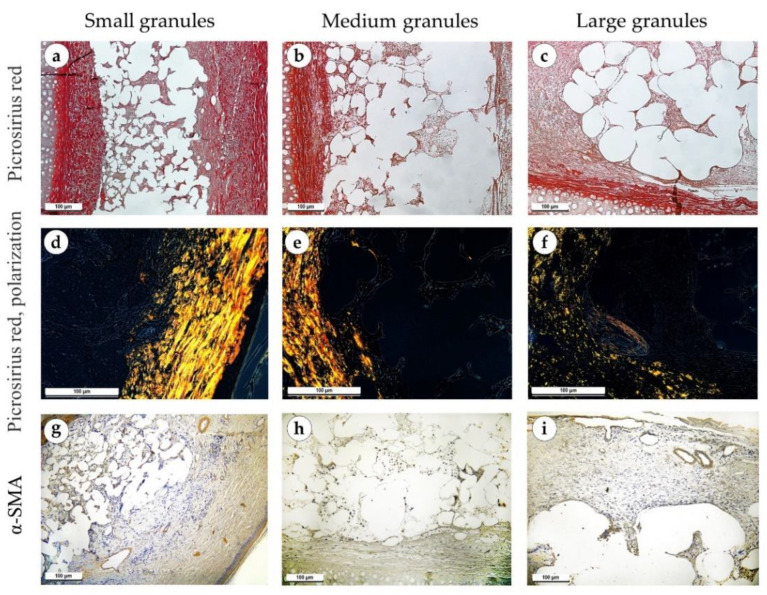
Sites of implantation of discs made of small (**a**,**d**,**g**), medium (**b**,**e**,**h**) and large (**c**,**f**,**i**) granules, 7 days after surgery (**a**–**c**) PSR stain, magnification ×100. (**d**–**f**) PSR stain, polarized light microscopy, magnification ×200. (**g**–**i**) immunohistochemical reaction with antibodies against α-SMA, magnification ×100. Scale bars are 100 µm.

One week after the operation, inflammatory processes prevailed over fibrosis. Necrosis of the skin was observed over the implanted material in some of the samples. There were no differences in morphological features between study groups. The tissues surrounding the implant were edematous in all samples. Granulation tissue with a high density of blood vessels grew between the elements of the implant, above and below it (Figure 4a–c). A small number of fibroblasts without signs of proliferation were present in the granulation tissue (Figure 4d–f). The peri-implant tissue was weakly infiltrated with singular neutrophils and contained focal hemorrhages. Specialized connective tissue stains (PSR and Mallory’s trichrome) highlighted loose and immature fibrous capsules (Figure 4g–I and Figure 5a–c). Polarized light microscopy of the slides revealed a greenish glow (weak anisotropy) of immature collagen fibers of the peri-implant tissue located under highly anisotropic resident dermis (Figure 5d–f). An immunohistochemical reaction with antibodies against α-SMA showed weak expression of this marker in isolated fibroblasts (+) (Figure 5g–i). 

**Figure 6:**
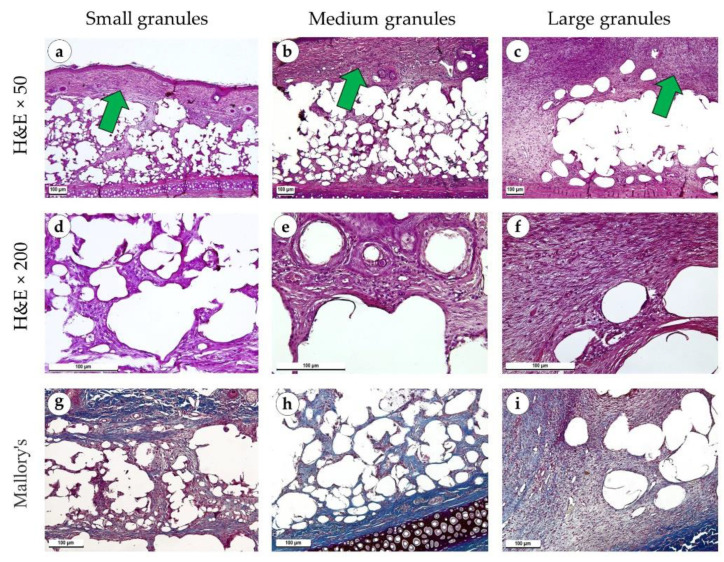
Sites of implantation of discs made of small (**a**,**d**,**g**), medium (**b**,**e**,**h**) and large (**c**,**f**,**i**) granules, 14 days after surgery. (**a**–**c**) H&E stain, magnification ×50. (**d**–**f**) H&E stain, magnification ×200. (**g**–**i**) Mallory’s trichrome stain, magnification ×100. Scale bars are 100 µm. Immature connective tissue capsules are marked with green arrows.

**Figure 7:**
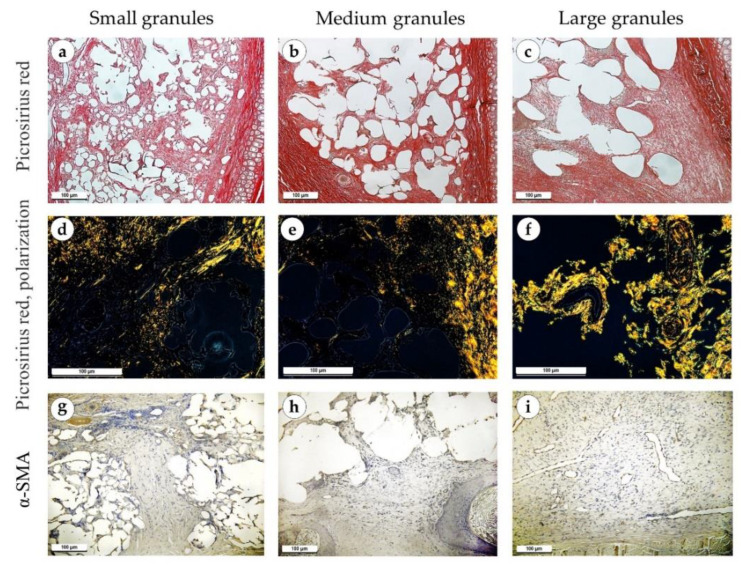
Sites of implantation of discs made of small (**a**,**d**,**g**), medium (**b**,**e**,**h**) and large (**c**,**f**,**i**) granules, 14 days after surgery. (**a**–**c**) PSR stain, magnification ×100. (**d**–**f**) PSR stain, polarized light microscopy, magnification ×200. (**g**–**i**) immunohistochemical reaction with antibodies against α-SMA, magnification ×100. Scale bars are 100 µm.

Two weeks after the operation, newly formed connective tissue contained numerous proliferating myo- and fibroblasts located between, below and above the implant (Figure 6a–c). In cases with small and medium granule implants, the infiltrate included macrophages and singular multinucleated giant cells in addition to lymphocytes (Figure 6d,e). The immune infiltration around large granular implants was insignificant (Figure 6f). It was represented by small clusters of lymphocytes. The capsule around the implants of small and medium granules consisted of parallelly oriented thick collagen fibers and was red when stained with PSR and blue with Mallory’s trichrome stains (Figure 6g,h and Figure 7a,b). Large granule implants were surrounded by a capsule formed by multidirectional bundles of collagen fibers and fibroblasts, which was visualized with specialized connective tissue stains (Figure 6i and Figure 7c). Under polarized light, the connective tissue in the capsule of implants with small and medium granules had a yellow glow, while the capsule around implants with large granules had a larger portion of green glow (Figure 7d–f). An immunohistochemical reaction with antibodies against α-SMA showed moderate expression of this marker in proliferating fibroblasts (++), especially in the area of the capsule around implants with small and medium granules (Figure 7g–i).

**Figure 8:**
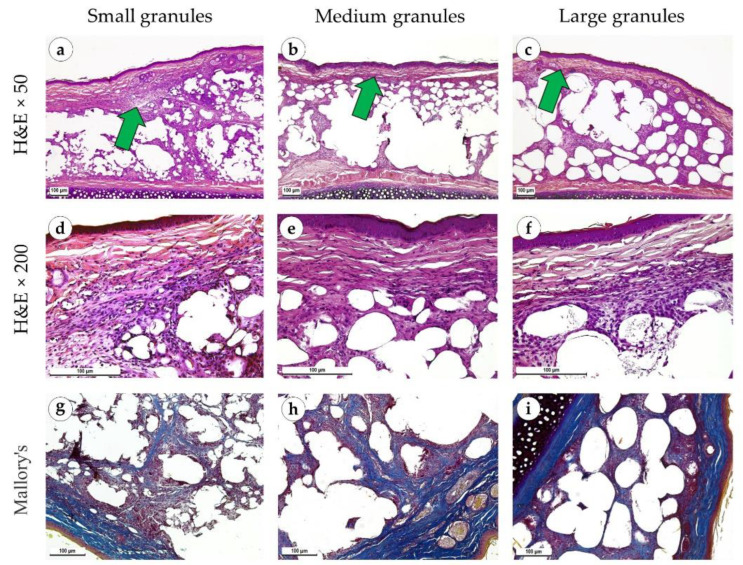
Sites of implantation of discs made of small (**a**,**d**,**g**), medium (**b**,**e**,**h**) and large (**c**,**f**,**i**) granules, 30 days after surgery. (**a**–**c**) H&E stain, magnification ×50. (**d**–**f**) H&E stain, magnification ×200. (**g**–**i**) Mallory’s trichrome stain, magnification ×100. Scale bars are 100 µm. Connective tissue capsules are marked with green arrows.

**Figure 9:**
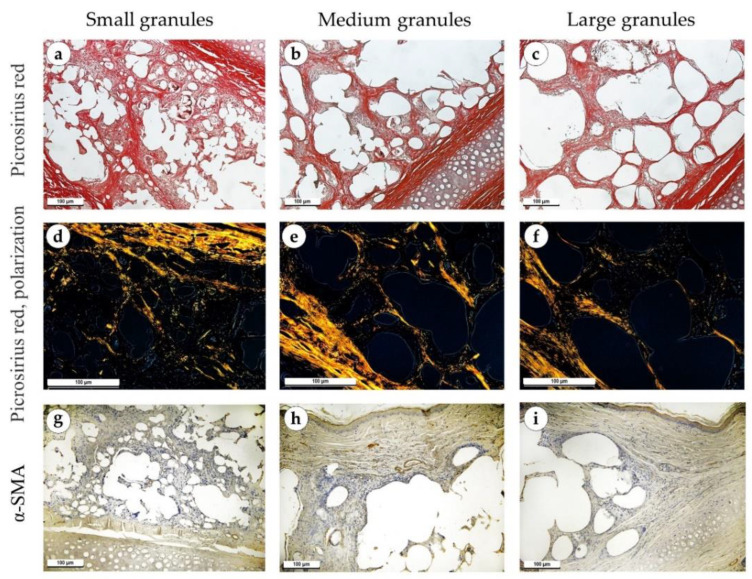
Sites of implantation of discs made of small (**a**,**d**,**g**), medium (**b**,**e**,**h**) and large (**c**,**f**,**i**) granules, 30 days after surgery. (**a**–**c**) PSR stain, magnification ×100. (**d**–**f**) PSR stain, polarized light microscopy, magnification ×200. (**g**–**i**) immunohistochemical reaction with antibodies against α-SMA, magnification ×100. Scale bars are 100 µm.

One month after the operation, the implant material granules were separated by diffuse thick layers of mature connective tissue (Figure 8a–c). Immune cell infiltration in the implants of small and medium granules was significant, represented by lymphocytes, macrophages, giant multinucleated cells and eosinophils (Figure 8d,e). The infiltration around the implants with large granules was relatively less intensive (Figure 8f). In some cases, there was a layer of connective tissue with signs of fibrosis between the perichondrium and the implant. The implants of small granules were surrounded by a thick fibrous capsule consisting of parallel collagen fibers (Figure 8g and Figure 9a). Actively proliferating fibroblasts were present in large numbers. In medium granule implants, the capsule was thinner and consisted of several layers of collagen fibers and proliferating fibroblasts (Figure 8h and Figure 9b). Around the implants with large granules, the capsule was immature and consisted of thin collagen fibers and bundles of fibroblasts, which was more evident in samples stained with PSR and Mallory’s trichrome stains (Figure 8i and Figure 9c). Under polarized light, the capsule above the implants of medium and small granules had a yellowish-orange glow, but the capsule around the large granules had a greenish-yellow glow (Figure 9d–f). An immunohistochemical reaction with antibodies against α-SMA revealed its strong expression in significantly proliferating fibroblasts in cases with small and medium granule implants (+++), while milder expression was observed in fibroblasts in the capsule around implants made of large granules (++) (Figure 9g–i).

**Figure 10:**
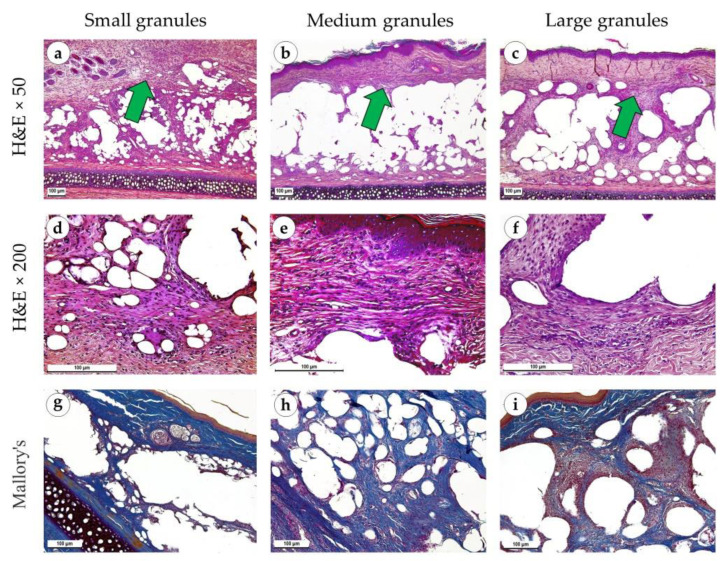
Sites of implantation of discs made of small (**a**,**d**,**g**), medium (**b**,**e**,**h**) and large (**c**,**f**,**i**) granules, 60 days after surgery. (**a**–**c**) H&E stain, magnification ×50. (**d**–**f**) H&E stain, magnification ×200. (**g**–**i**) Mallory’s trichrome stain, magnification ×100. Scale bars are 100 µm. Connective tissue capsules are marked with green arrows.

**Figure 11:**
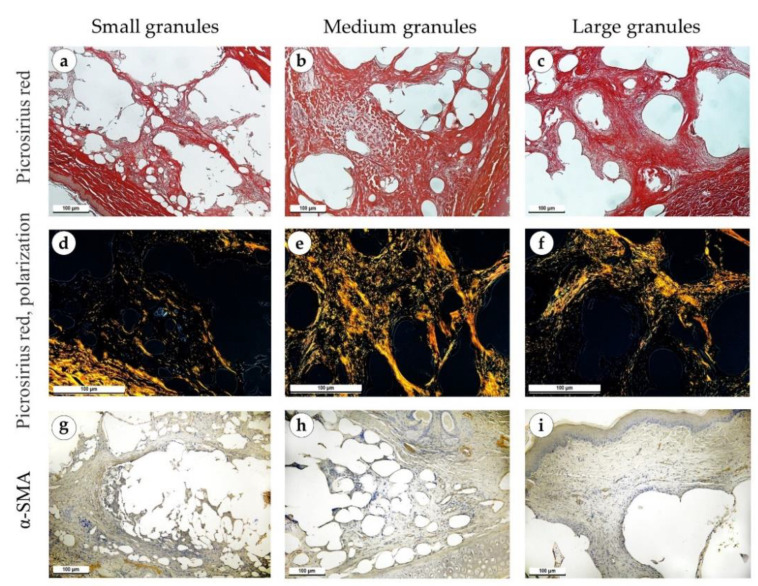
Sites of implantation of discs made of small (**a**,**d**,**g**), medium (**b**,**e**,**h**) and large (**c**,**f**,**i**) granules, 60 days after surgery. (**a**–**c**) PSR stain, magnification ×100. (**d**–**f**) PSR stain, polarized light microscopy, magnification ×200. (**g**–**i**) immunohistochemical reaction with antibodies against α-SMA, magnification ×100. Scale bars are 100 µm.

Two months after the operation, the connective tissue was thick and fibrotic around the implant of small and medium granules compared to the thinner capsules around the implant of large granules (Figure 10a–c). Immune infiltration around the implants made of small and medium granules was significantly expressed and represented by lymphocytes, macrophages and clusters of giant multinucleated cells (Figure 10d,e). Infiltration was weaker around the implants made of large granules (Figure 10f). The fibrous capsule surrounding the small granule implants consisted of parallelly oriented collagen fibers and included myofibroblasts (Figure 10g and Figure 11a). In the samples made of medium granules, the capsule was thicker and consisted of several layers of parallelly oriented collagen fibers with myofibroblasts (Figure 10h and Figure 11b). Large granule implants were surrounded by a less mature capsule consisting of thin collagen fibers and bundles of fibroblasts (Figure 10i and Figure 11c). Under polarized light, the connective tissue and capsule around small and medium granule implants had a bright orange glow, while the capsule around the large granule implants had a yellowish glow (Figure 11d–f). An immunohistochemical reaction with antibodies against α-SMA revealed its moderate expression in proliferating fibroblasts around the implants of large granules (++) (Figure 11i). We noticed expression in singular fibroblasts around the implants of small and medium granules, which was associated with a slowdown in proliferative and reparative processes and the formation of a stable and significantly fibrosed connective tissue (+) (Figure 11g,h).

**Figure 12:**
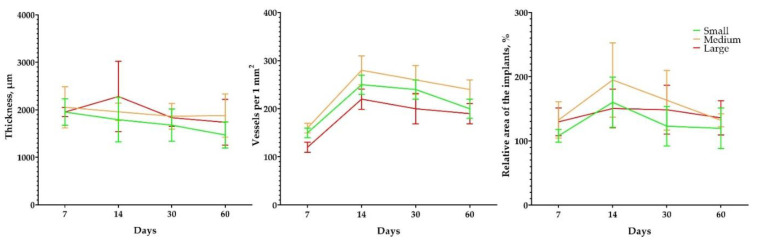
Morphometric graphs. Thickness of the peri-implant complex, µm at different time points. Density of vascularity in the peri-implant capsule, number of vessels per 1 mm^2^. Relative area of implants, % of the initial area. Mean values ± SD.

The data from the measurements of the thickness of the peri-implant tissue did not present statistical differences (Figure 12), with the highest values recorded for the group with implants made of large granules on day 14 (Table A11 in Appendix A). The density of vascularization in the peri-implant capsule and the relative area of the implants peaked on day 14 in all groups and were more pronounced in the group with implants made of medium-diameter granules (Table A12 in Appendix A). In all groups, we observed an increase in implants in relation to their original area, which amounted to more than 100%. At all time points except 60 days, we observed the highest values in the group with samples implanted from medium granules (Table A13 in Appendix A).

**Figure 13:**
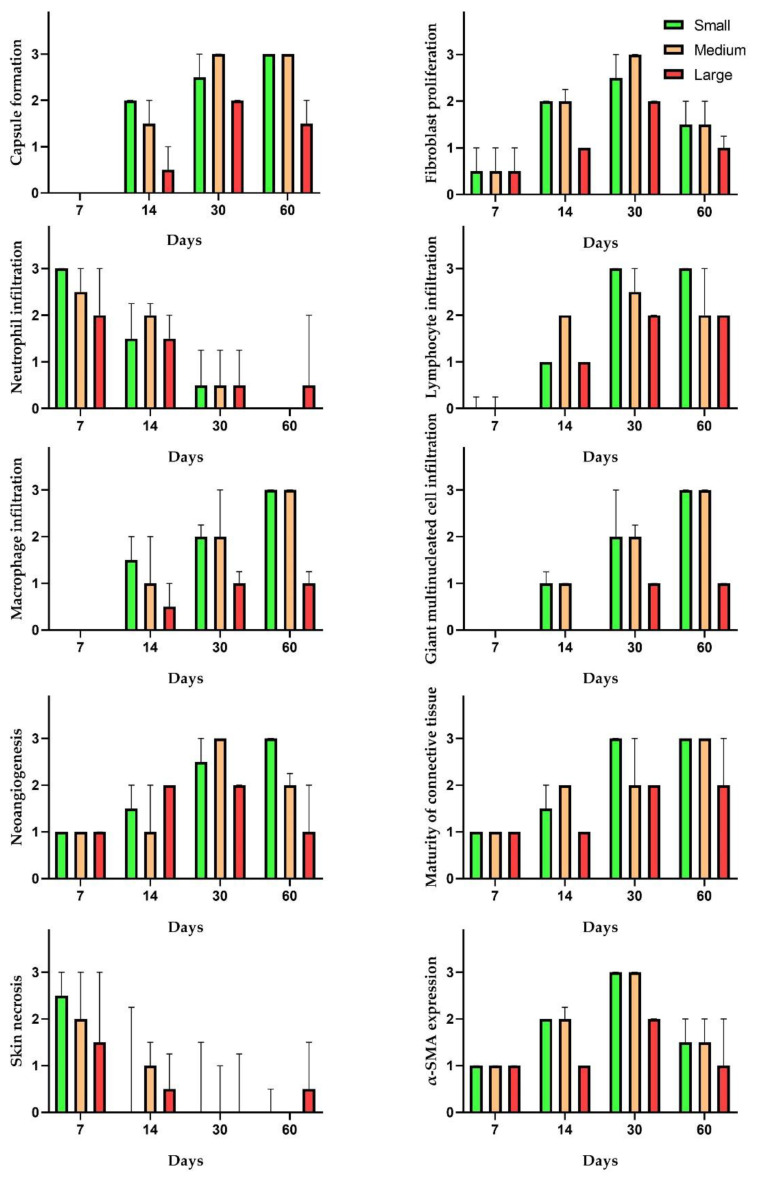
Semi-quantitative analysis of morphological features. Median values ± interquartile range.

Tissues in the areas of implantation of scaffolds made of small and medium granules had significant differences in a number of morphological features (capsule formation, fibroblast proliferation, macrophage and giant multinucleated cell infiltration, neoangiogenic and α-SMA expression) in comparison to the tissues in the group of large granules (Figure 13). Fibroblast proliferation differed between these groups at day 14 after the implantation while the difference in capsule formation was identified at day 60. While there were no differences in neutrophil and lymphocyte infiltration, macrophages and giant multinucleated cells infiltrated peri-implant tissues significantly more in the groups with small and medium granules since day 14. Peri-implant tissues around scaffolds made of small granules had the strongest stimulation impact on neoangiogenesis. There was an increase in the number of α-SMA positive cells in tissues around the implants made of small and medium granules at days 14 and 30.

### 3.2. Optical Coherence Tomography

**Figure 14:**
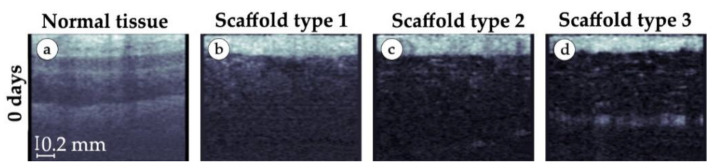
OCT scans of tissue samples in vivo. (**a**) Tissue without implant. (**b**) Tissue with implant made of small granules. (**c**) Tissue with implant made of medium granules. (**d**) Tissue with implant made of large granules.

On the day of implantation, we visualized a clear lower epidermal border (Figure 14b–d). The border was mostly smooth with slight curves. The structure of the skin (Figure 14a) was strongly disturbed compared to the image of tissue without an implant; in the cases of small and medium granule implants (Figure 14b,c), the dermis was not visualized. A possible explanation of these changes is the presence of biological fluids in the area between the epidermis and the implant. In the areas with large granule implants, there was a noticeable border, presumably the upper edge of the implant (Figure 14d), the size of the granules of which was significantly larger than the size of the granules of implants of types 1 and 2 (small and medium granules).

**Figure 15:**
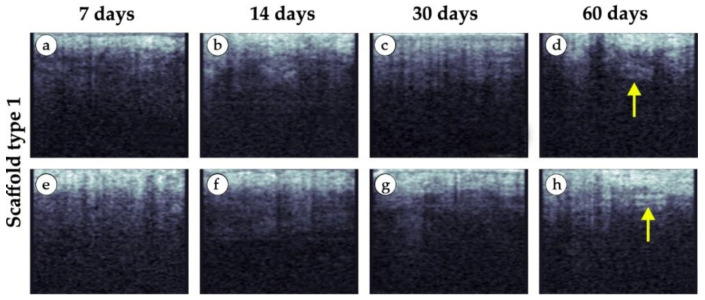
Representative examples of OCT scans of tissue samples in vivo (implant made of small granules). (**a**,**e**) 7 days after implantation. (**b**,**f**) 14 days after implantation. (**c**,**g**) 30 days after implantation. (**d**,**h**) 60 days after implantation. Layer of dense connective tissue was detected at day 60 (yellow arrows).

Specific changes in the tissue structure in the area of implantation of small granule implants are shown in the representative images in Figure 15. In contrast to the day of implantation, a clear lower boundary of the epidermis was not visualized. The blurring of the border indicated changes in the epidermis. At 60 days, visualization of a thin layer under the epidermis was typical (yellow arrows in Figure 15d,h). Presumably, the layer was formed by a fibrous capsule (see Figure 10a).

**Figure 16:**
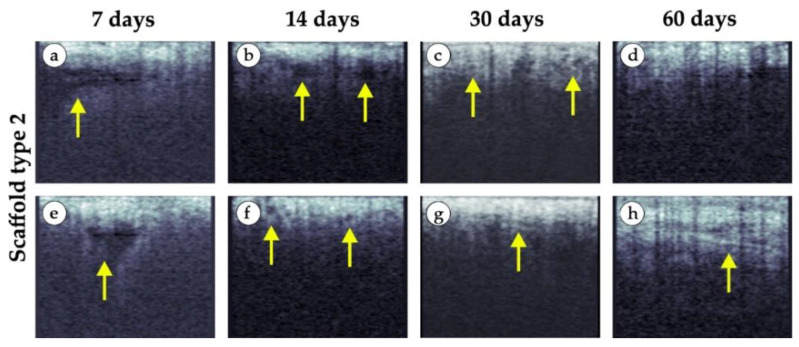
Representative examples of OCT scans of tissue samples in vivo (implant made of medium granules). (**a**,**e**) 7 days after implantation. (**b**,**f**) 14 days after implantation. (**c**,**g**) 30 days after implantation. (**d**,**h**) 60 days after implantation. Areas of edema at days 7 and 14 (yellow arrows). Layer of connective tissue was detected at day 30 and 60 (yellow arrows).

Specific changes in the tissue structure in the area of implantation of medium granule implants are shown in the representative images in Figure 16. Areas of edema (fluid inclusions) remained in most cases on the 7th day (yellow arrows in Figure 16a,e) and it was weaker on the 14th day (yellow arrows in Figure 16b,f). After 14 and 30 days, in some cases, we visualized individual small inclusions like cysts (yellow arrows in Figure 16c,f). These inclusions were almost absent on the 60th day, but in some cases, a thin layer could be seen in the tissue structure (yellow arrow in Figure 16h), indicating, presumably, the formation of a fibrous capsule. This layer was less expressed on the 30th day (yellow arrow in Figure 16g).

**Figure 17:**
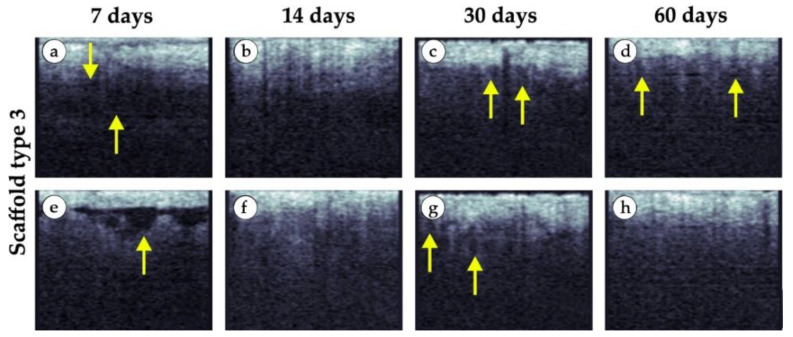
Representative examples of OCT scans of tissue samples in vivo (implant made of large granules). (**a**,**e**) 7 days after implantation. (**b**,**f**) 14 days after implantation. (**c**,**g**) 30 days after implantation. (**d**,**h**) 60 days after implantation. 60 days after implantation. Areas of edema at day 7 (yellow arrows). Signs of granular material were detected at day 30 and 60 (yellow arrows).

Specific changes in the tissue structure in the area with large granule implants are shown in the representative images in Figure 17. We noticed areas of edema (fluid inclusions) in most cases on the 7th day (yellow arrows in Figure 17a,e). After 14 days, the lower border of the epidermis was not visualized (Figure 17b,f). After 30 days, we saw small round inclusions (yellow arrows in Figure 17c,g), which could be cysts or implant granules. A few granular tissue structures under the epidermis persisted after 60 days (Figure 17d). In contrast to tissue images obtained in the area of implantation of scaffolds of the first and second types (small and medium granules), for the implantation of the scaffold made of large granules, after 60 days we did not notice visualization of an additional thin layer (fibrous capsule).

**Figure 18:**
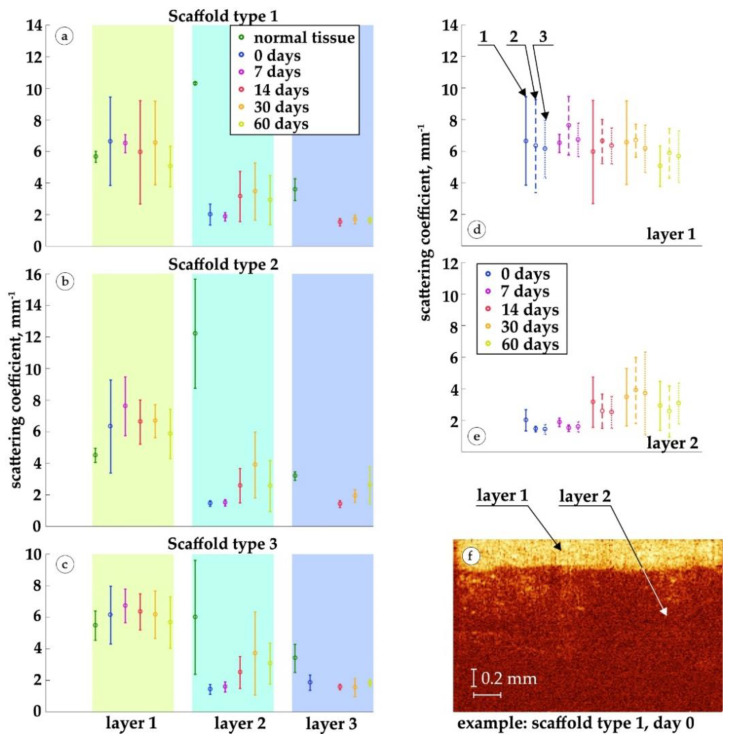
Analysis of the scattering coefficient of selected tissue layers on OCT images. (**a**–**c**) Changes over time of the scattering coefficient of three tissue layers during implantation of implants of the small, medium and large granules. (**d**,**e**) Comparison of scattering coefficients of the first and second layers, respectively, for implants of the small granules (marked 1), medium granules (2) and large granules (3). (**f**) An example of the original OCT B-scan.

The results of the analysis of the scattering coefficient in the implantation zones are shown in Figure 18.

The significant variance of the scattering coefficient is explained by large structural differences between tissue samples within each sample. For all types of implants, we distinguished from OCT images at least three layers of tissue (Figure 18a–c). The first layer, the epidermis, is characterized by an increase in the scattering coefficient on the day of implantation in comparison with that of the epidermis without implantation, as well as a gradual decrease in the value of the scattering coefficient of the epidermis on subsequent days. This trend continues for all types of implants. The rise of the scattering coefficient is associated with an increase in tissue heterogeneity. 

The second layer is also characterized by the same alteration of the scattering coefficient: a sharp decrease on the day of implantation and a subsequent increase over 30 days, which describes the growth of the heterogeneity of the second layer (dermis). We noticed a slight decrease in the scattering coefficient on the 60th day.

Changes in the scattering coefficient of the third layer were not significant. However, in contrast to implants of the small and large granules, for the implant of the medium granules, an increase in the scattering coefficient from 14 to 60 days was noticeable.

A comparative analysis of the scattering coefficient for the first and second layers of three types of implants is shown in Figure 18d,e. We noticed that after 7, 14, 30 and 60 days, the scattering coefficient of the first layer for the medium granules implant was slightly higher than that for the implants made of small and large granules. However, these changes lie within the variance.

## 4. Discussion

In this study, we investigated the effect of the microtextural profile of implants on the fibrotic and inflammatory responses of the recipient organism. For this purpose, three different types of implants consisting of polymer granules of different diameters were placed in the skin pocket model of the rabbit ear.

The animal model used in this work is a modification of the approach used to model hypertrophic scarring based on the ischemic nature of rabbit ear skin wounds [20,22]. Implants were placed in the wound defects in the form of a pocket between the dermis and the perichondrium of the ear cartilage. This model helped to simulate fibrotic and inflammatory responses in the initial profibrotic tissue niche. The major benefits of choosing this model are reproducibility of the tissue reaction to biomaterials (including morphometric measurements) and fast formation of mature connective tissue capsules [21]. 

The implants were fabricated by surface-selective laser sintering from PDL04 polylactide granules of different diameters (1–56 µm, 56–100 µm and 100–200 µm) and had a microtextured surface.

Polylactide is one of the most promising biopolymers produced from non-toxic renewable resources. Due to its properties such as biocompatibility, biodegradability, mechanical strength and manufacturability, polylactide is actively used in biomedicine as a raw material for implants, surgical sutures, drug delivery systems and other tissue engineering structures. This material is also actively used in dentistry, cardiovascular surgery, traumatology, orthopedics, dermatology and cosmetology [23,24,25,26,27]. The choice of PLA for the present study was based on its pro-fibrotic effects accelerating the formation and maturation of peri-implant connective tissue capsules [21].

**Figure 19:**
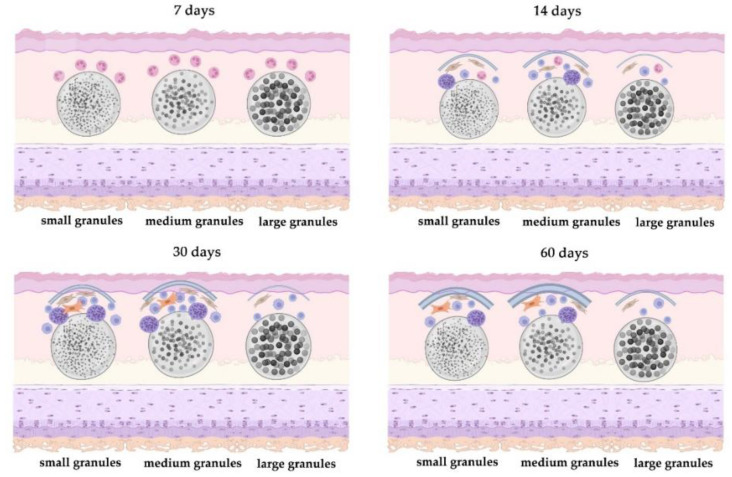
Graphical scheme of the impact of the granule size on the tissue reaction.

Immune cell reaction was the major tissue reaction in all study groups during the first days of the implantation. The first intergroup differences were observed two weeks after the operation when fibroblast proliferation and immune cell infiltration began to be more pronounced around the implants of small and medium granules. The immature capsule formed by the end of the first month and it was thicker around the small and medium granule implants. These capsules became thick and mature by the end of the second month indicating that small and medium granules facilitated and accelerated the fibrosis (Figure 19).

Histological, morphometric and immunohistochemical investigations showed that microtextured implants from small (1–56 μm) and medium (56–100 μm) granules induced more active inflammatory processes and potentiated the growth of the connective tissue capsule. These results correlate with the study in which silicone implants with different textures were implanted in pigs for 9 months. The study showed that textured implants induced the formation of connective tissue capsules with less pronounced contractures than implants with a smooth surface. Capsular pressure was also higher in the group of implants with a smoother surface [16,28]. The research group of Robert Langer examined the foreign body response and capsular fibrosis around miniaturized or full-scale clinically approved breast implants with different surface topography (average roughness, 0–90 μm) for up to one year after the implantation in the mammary fat pads of mice or rabbits. It means that while minor topography is better than a completely smooth implant surface, it should be limited and should not create a complicated area of contact between the foreign body and soft tissues [16]. We can compare these results of high biocompatibility of minor texture to our large granule implants that were close to smooth but still had a slight texture. Potentially, this proves that the fibrotic component of tissue response to both PLA and silicone develops in the same trajectory.

Atlan M. et al. implanted discs with a diameter of 30 mm, cut from commercial breast implants with different microtextural surfaces, subcutaneously in the rats for 6 weeks. The morphology of the capsule was similar in all groups, and in cases of implants with larger textures, a disorganized arrangement of collagen fibers was observed. The tissue around fine texture implants was approximately the same in thickness in all areas and the collagen fibers of the capsule were parallel and unidirectional. However, in the groups with larger textures, there were small “protrusions” on the surface of the implants, resulting in a slight disorganization of the collagen fibers in the peri-implant capsule. Also, the authors, in their publication, evaluated the maximum force required to separate the capsule from implants with different surface textures using a peel test in the sixth week after surgery. As the surface texture became more complex, there was an increase in the peak force required to separate the surrounding tissue from the implant [29]. Another study focused on the analysis of inflammatory and fibrotic reactions associated with implantation of samples with different surface profiles (smooth, micro- and macrotextural). The number of myo- and fibroblasts in immunohistochemical reactions with antibodies against α-SMA and vimentin was approximately the same in all groups. The severity of fibrotic changes, collagen expression and the thickness of the peri-implant capsule were the lowest in cases with implanted microtexture samples [30].

OCT is a non-invasive method of examining thin layers of skin and mucous membranes, blood vessels, eye and dental tissues. The physical principle of operation of OCT is similar to ultrasound, with the only difference being that OCT uses optical radiation in the near infrared range (~1 µm) rather than acoustic waves to probe biological tissue. In dermatology, OCT can be used for investigations of skin cancer, inflammatory diseases and wound healing and applied as an assistance for planning and controlling various treatments, such as follicular unit extraction and drug delivery through nails and skin [31,32].

The OCT data coincided with our results obtained during histological examination. The scattering coefficient of the first layer for the medium granule implant was slightly higher than that for the implants made of small and large granules. In cases with implants made of small and medium granules, we observed the formation of a thick peri-implant capsule on OCT scans and microscopy. While this study finds OCT useful for the non-invasive evaluation of the peri-implant reaction, low detalization of morphological structures and the need for post-analysis processing limit its practical application. Further development of OCT technology will possibly make it valuable in assessing biocompatibility of subcutaneous implants such as Implanon or Nexplanon that were shown to be able to migrate from the implantation site. 

In summary, our results confirm the hypothesis that inflammatory and fibrotic reactions are weak in cases of implantation of samples with a close to smooth surface. In our experiment, these implants were prepared from large polymer powder granules with diameter 100–200 µm. Large granule implants had spaces between polymer granules. We believe that these rough surfaces of the implants made it difficult for macrophages and other immune cells to adhere on it. As a result, the recipient’s immune response is not so active in these cases. Implants made of small and medium-sized granules were closest in their characteristics to textured implants and induced a more pronounced inflammatory response in the form of lymphocyte and macrophage infiltration with admixture of giant multinucleated cells. This lead to more expressive fibrotic processes, hyperplasia and hypertrophy of fibroblasts, their transformation into myofibroblasts, active synthesis of collagen fibers and the formation of a dense connective tissue peri-implant capsule around the material. 

The formation of a capsule is a normal reaction of the recipient tissue to the implant, but sometimes this reaction can be excessive and lead to such complications as contracture of the capsule (compression of the capsule around the implanted material) or even to the formation of a double capsule due to the developing seromas [33,34]. The etiology of contracture development is not fully understood; possible causes are the position of the wound area, hypertrophic scars, biofilm formation, excessive inflammatory response leading to the formation of a large number of myo- and fibroblasts and abundant collagen synthesis [35]. Systematic reviews have shown that the texturing of implants reduces the incidence of early capsular contracture [36]. It is also believed that the texture of the implant surface can affect the formation of the peri-implant capsule, the organization of collagen fibers and the adhesive properties of the implant [37,38,39,40]. When a surgeon uses an implant with a smooth surface, a dense capsule with unidirectional organized collagen fibers is formed around it and the adhesive properties of its surface are reduced, which can lead to implant migration [41,42,43]. When implanting samples with a rough macrotextural surface, the depth and complexity of the relief may result in ingrowth of connective tissue into the upper layers of the implant [28,43]. To reduce fibrotic reactions and the risk of capsular contracture, nano- and microtextured implants have been developed which have the characteristics of both smooth and rough surfaces [43]. The microtextured topography of the implant surface has been shown to contribute to a low rate of capsular contracture, with consistency of technique and type of implant used by the same surgeon [44]. In addition, it is also worth noting that much attention is paid to surface modification in combination with drug delivery as a prevention of contracture of the implant capsule [21,33,45,46,47].

The present study has several limitations. Firstly, while the experimental model of peri-implant fibrosis offers high reproducibility, it does not permit direct translation of findings compared to more orthotopic models like pig breast implantation. This raises the possibility that the microtexture of implants may have a different impact on glandular and adipose tissues, which are more abundant in the breast than in the rabbit ear. Secondly, our focus was solely on microtextured solid synthetic implants, excluding considerations of nanotexture or the risks associated with inner compartment gel exposure to surrounding tissues. Thirdly, although the OCT analysis provided promising insights, it did not yield conclusive results regarding changes in the soft tissues at the implantation site. Additional limitations include the implant’s simple macrostructure, the absence of considerations of shear stress induced by fluid flow and a lack of mechanical testing for general surface characterization (e.g., via atomic force microscopy). These gaps highlight areas for future research in the domain of implant-tissue biology.

## 5. Conclusions

In conclusion, this research underscores the importance of fine textures for biomaterials that have been shown to be effective across different models. Microtextured implants from large granules led to weak inflammatory processes and less accumulation of giant multinucleated cells in peri-implant tissues, resulting in milder growth of the connective tissue capsule. These findings have a potential to be translated into several biomedical applications including the design of implants and scaffolds for tissue engineering, guided tissue regeneration and the development of ex vivo models (“organs-on-chip”). This work draws attention to the understudied fields of matrix biology and biomimetics. Further investigations into the communication between fibroblasts and giant multinucleated cells may reveal new targets for pharmaceutical control over inflammation and regeneration. 

## Data Availability

The relevant data generated and (or) analyzed in the current study are available from the corresponding author upon reasonable request.

## Appendix A

**Table A1:** Criteria of semi-quantitative histological scoring of the capsule formation.

Criteria	
Score	Capsule Formation
0	No signs of capsule formation
1	Immature capsule of thin bundles of collagen fibers
2	Moderately mature capsule of several layers of collagen fibers
3	Mature capsule of thick fibrous collagen fibers

**Table A2:** Criteria of semi-quantitative histological scoring of fibroblast proliferation.

Criteria	
Score	Fibroblast Proliferation
0	No signs of fibroblast proliferation
1	Weak signs of hypertrophy and hyperplasia of fibroblasts, an increase in their density by less than 10%
2	Moderate signs of hypertrophy and hyperplasia of fibroblasts, an increase in their density by 20–30%
3	Pronounced signs of hypertrophy and hyperplasia of fibroblasts, an increase in their density by more than 30%

**Table A3:** Criteria of semi-quantitative histological scoring of infiltration by neutrophils.

Criteria	
Score	Infiltration by Neutrophils
0	No neutrophil infiltration
1	Singular neutrophils in the tissue (less than 10 in the field of view at ×400 magnification)
2	A moderate number of neutrophils in the tissue (from 11 to 29 in the field of view at ×400 magnification)
3	A large number of neutrophils in the tissue (more than 30 in the field of view at ×400 magnification)

**Table A4:** Criteria of semi-quantitative histological scoring of infiltration by lymphocytes.

Criteria	
Score	Infiltration by Lymphocytes
0	No lymphocyte infiltration
1	Singular lymphocytes in the tissue (less than 10 in the field of view at ×400 magnification)
2	A moderate number of lymphocytes in the tissue (from 11 to 29 in the field of view at ×400 magnification)
3	A large number of lymphocytes in the tissue (more than 30 in the field of view at ×400 magnification)

**Table A5:** Criteria of semi-quantitative histological scoring of the infiltration by macrophages.

Criteria	
Score	Infiltration by Macrophages
0	No macrophage infiltration
1	Singular macrophages in the tissue (less than 5 in the field of view at ×400 magnification)
2	A moderate number of macrophages in the tissue (from 6 to 15 in the field of view at ×400 magnification)
3	A large number of macrophages in the tissue (more than 16 in the field of view at ×400 magnification)

**Table A6:** Criteria of semi-quantitative histological scoring of the infiltration by giant multinucleated cells.

Criteria	
Score	Infiltration by Giant Multinucleated Cells
0	No infiltration by giant multinucleated cells
1	Singular giant multinucleated cells in the tissue (less than 2 in the field of view at ×400 magnification)
2	A moderate number of giant multinucleated cells in the tissue (from 3 to 6 in the field of view at ×400 magnification)
3	A large number of giant multinucleated cells in the tissue (more than 7 in the field of view at ×400 magnification)

**Table A7:** Criteria of semi-quantitative histological scoring of the neoangiogenesis.

Criteria	
Score	Neoangiogenesis
0	No signs of newly formed blood vessels
1	The beginning of vessel formation: the vascular wall is absent; the endothelium is represented by a thin layer of endotheliocytes
2	Continued vessel formation: t. Adventitia is absent, muscle fibers in t. Media are thin, the endothelium is of normal structure
3	The blood vessels are formed: the wall has a three layer structure (t. Adventitia, Media, Intima), the endothelium is of normal structure

**Table A8:** Criteria of semi-quantitative histological scoring of the maturity of connective tissue.

Criteria	
Score	Maturity of Connective Tissue
0	No newly formed connective tissue around the implant
1	Granulation tissue with a lot of blood vessels
2	Connective tissue consisting of bundles of thin collagen fibers
3	The connective tissue is mature, made of bundles of fibrous thick collagen fibers

**Table A9:** Criteria of semi-quantitative histological scoring of the skin necrosis.

Criteria	
Score	Skin Necrosis
0	No necrosis in the skin
1	Small areas of necrosis in the upper layers of the epithelium
2	Skin necrosis of medium area in all layers of the epithelium
3	Necrosis of extensive size, penetrating into the upper layers of the dermis

**Table A10:** Criteria of semi-quantitative histological scoring of the IHC staining for α-SMA in peri-implant tissue.

Criteria	
Score	Expression of α-SMA
0/-	Absence of the staining
1/+	Individual positive cells
2/++	Thin (≤30 µm) continuous layer of positive cells
3/+++	Thick (>30 µm) continuous layer of positive cells

**Table A11:** Thickness of the peri-implant complex, nm (mean value with standard deviation).

Implants			
Days	Small Granules	Medium Granules	Large Granules
7	1953.2 ± 279.2	2054 ± 434.8	1952.5 ± 90.4
14	1792 ± 467.6	1957.2 ± 185.1	2280.7 ± 405.7
30	1677 ± 340.5	1865.3 ± 270.4	1834.6 ± 172.3
60	1470.5 ± 274.5	1878.1 ± 454.7	1736.4 ± 459.1

**Table A12:** Density of vascularity in the peri-implant capsule (number of vessels/mm^2^) (mean value with standard deviation).

Implants			
Days	Small Granules	Medium Granules	Large Granules
7	150 ± 10	160 ± 10	120 ± 10
14	250 ± 20	280 ± 30	220 ± 20
30	240 ± 20	260 ± 30	200 ± 30
60	200 ± 20	240 ± 20	190 ± 20

**Table A13:** Relative area of implants, % of the original area.

Implants			
Days	Small Granules	Medium Granules	Large Granules
7	108 ± 9.89	132.65 ± 28.21	129.7 ± 20.59
14	160.16 ± 39.04	195 ± 57.84	150.5 ± 28.83
30	123 ± 30.79	163.4 ± 46.41	148.51 ± 36.31
60	119.7 ± 31.49	132.06 ± 10.08	136 ± 25.57
